# Circulating Biomarkers in Pulmonary Arterial Hypertension: An Update

**DOI:** 10.3390/biom14050552

**Published:** 2024-05-03

**Authors:** Michele Correale, Lucia Tricarico, Ester Maria Lucia Bevere, Francesco Chirivì, Francesca Croella, Paolo Severino, Valentina Mercurio, Damiano Magrì, Frank Dini, Roberto Licordari, Matteo Beltrami, Giuseppe Dattilo, Andrea Salzano, Alberto Palazzuoli

**Affiliations:** 1 Cardiothoracic Department, Ospedali Riuniti University Hospital, 71100 Foggia, Italy; 2 Department of Medical and Surgical Sciences, University of Foggia, 71100 Foggia, Italy; lucia.tricarico.lt@gmail.com (L.T.); esterb94@hotmail.it (E.M.L.B.); francesco.chirivi11@gmail.com (F.C.); 3 Cardiothoracic Vascular Department, Division of Provincial Cardiology, Santissima Annunziata Hospital and Delta Hospital, Azienda Unità Sanitaria Locale di Ferrara, 44121 Ferrara, Italy; fracroella@gmail.com; 4 Department of Clinical, Internal, Anesthesiology and Cardiovascular Sciences, Sapienza University of Rome, Viale del Policlinico, 00185 Rome, Italy; paolo.severino@uniroma1.it; 5 Department of Translational Medical Sciences, Federico II University, 80138 Naples, Italy; valemercurio@yahoo.com; 6 Department of Clinical and Molecular Medicine, Azienda Ospedaliera Sant’Andrea, “Sapienza” Università degli Studi di Roma, 00161 Rome, Italy; damiano.magri@uniroma1.it; 7 Istituto Auxologico IRCCS, Centro Medico Sant’Agostino, Via Temperanza, 6, 20127 Milan, Italy; franklloyddini@gmail.com; 8 Department of Public Health and Clinical Medicine, Umeå University, 901 87 Umeå, Sweden; 9 Department of Biomedical and Dental Sciences and Morphofunctional Imaging, Section of Cardiology, University of Messina, 98122 Messina, Italy; robertolicordari@gmail.com (R.L.); dattimed@hotmail.it (G.D.); 10 Arrhythmia and Electrophysiology Unit, Careggi University Hospital, 50134 Florence, Italy; beltrami.matteo1@gmail.com; 11 Cardiology Unit, AORN A Cardarelli, 80131 Naples, Italy; andre.salzano@gmail.com; 12 Cardiovascular Diseases Unit, Cardio-Thoracic and Vascular Department, S. Maria alle Scotte Hospital, University of Siena, 53100 Siena, Italy; palazzuoli2@unisi.it

**Keywords:** biomarker, pulmonary arterial hypertension, pulmonary hypertension, diagnosis, treatment

## Abstract

Pulmonary arterial hypertension (PAH) is a rare subtype of group 1 pulmonary hypertension (PH) diseases, characterized by high pulmonary artery pressure leading to right ventricular dysfunction and potential life-threatening consequences. PAH involves complex mechanisms: vasoconstriction, vascular remodeling, endothelial dysfunction, inflammation, oxidative stress, fibrosis, RV remodeling, cellular hypoxia, metabolic imbalance, and thrombosis. These mechanisms are mediated by several pathways, involving molecules like nitric oxide and prostacyclin. PAH diagnosis requires clinical evaluation and right heart catheterization, confirming a value of mPAP ≥ 20 mmHg at rest and often elevated pulmonary vascular resistance (PVR). Even if an early and accurate diagnosis is crucial, PAH still lacks effective biomarkers to assist in its diagnosis and prognosis. Biomarkers could contribute to arousing clinical suspicion and serve for prognosis prediction, risk stratification, and dynamic monitoring in patients with PAH. The aim of the present review is to report the main novelties on new possible biomarkers for the diagnosis, prognosis, and treatment monitoring of PAH.

## 1. Introduction

One of the most commonly used non-invasive and cost-effective approaches to evaluating patients is the use of biomarkers. 

To date, the Biomarkers and Surrogate Endpoint Working Group defines “biological markers (biomarkers)” as follows: a biomarker is objectively measured and evaluated as an indicator of normal biological processes, pathogenic processes, or pharmacologic responses to a therapeutic intervention [1]. As a result, a biomarker can derive from blood, genetic samples, urine, physiologic tests, imaging, and tissue biopsies [1].

According to the biomarker’s classification of the FDA-NIH Biomarker Working Group, there are different types of biomarkers, based on their main clinical role, i.e., diagnostic, monitoring, pharmacological response, predictive, prognostic, safety, and risk biomarkers. Each type of biomarker may provide information about the disease under consideration and potentially contribute to improving diagnosis, prognosis, and clinical outcomes. Furthermore, biomarkers may increase the knowledge about the mechanisms underlying the disease and, consequently, help the identification of potential new pharmacological targets.

An ideal biomarker should have the following characteristics: 1. high sensitivity and specificity; 2. be easy to obtain/collect and measure; 3. display a wide availability; 4. no/very low invasiveness; 5. be a clear sign of disease activity (able to predict risk stratification, treatment responsiveness, and clinical worsening) or a treatment target. About the quality of biomarkers, for a good biomarker to be considered useful, it should fulfill three requirements: 1. it should be widespread and accessible; 2. it should be modifiable with disease progression and treatment; 3. its measurement should be reproducible. In the present paper, on the basis of these three statements, the authors formed their judgment on the quality of the biomarkers currently available.

The testing method for a biological marker is very important. Biomarker testing is a way to look for genes, proteins, and other substances (called biomarkers) that can provide information about a disorder.

Biomarker tests have many different uses in clinical practice, including disease screening, diagnosis, treatment and post-treatment monitoring, and prognosis for estimating risk or time to clinical outcomes. In addition, biomarker tests are used to predict a patient response to specific treatments.

Such predictive biomarker tests are used by physicians to tailor treatment to an individual patient’s clinical condition and treatment goals. A subset of these tests includes biomarker tests for specific aberrations in biological mechanisms of action that are associated with response or resistance to a specific targeted therapy. The clinical use of these predictive tests may be in the design of molecularly targeted therapies.

Pulmonary hypertension (PH) is a disorder characterized by a mean pulmonary arterial pressure (mPAP) higher than 20 mmHg at rest. On the basis of hemodynamic characteristics (i.e., pulmonary vascular resistance—PVR—and pulmonary arterial wedge pressure—PAWP) and etiological factors (e.g., pulmonary diseases, left ventricle diseases), five groups can be identified; when pre-capillary PH (i.e., mPAP > 20 mmHg, PAWP ≤ 15 mmHg, and PVR > 2 WU) is present, without evidence of any other causes, pulmonary arterial hypertension (PAH) is diagnosed [2]. About PAH management, detailed clinical assessment and a few additional diagnostic tests are required. Despite biochemistry, echocardiography, lung imaging, and pulmonary function tests providing useful data [3], right heart catheterization is still essential for PAH diagnosis. Notably, PAH still lacks effective biomarkers to assist in its diagnosis and prognosis.

Several mechanisms are involved in PAH pathophysiology, including vasoconstriction, vascular remodeling, endothelial dysfunction of the pulmonary circle, inflammation, oxidative stress, cardiac fibrosis, pathological RV remodeling, cellular hypoxia, metabolic imbalance, and thrombosis. The molecular and cellular changes in the presence of PAH are complex and involve numerous signaling pathways, such as the nitric oxide and prostacyclin pathways, and related biomarkers [4].

Because specific therapies for PAH patients [5] are available, a timely and accurate diagnosis of PAH is critical for effective therapeutic intervention. However, clinical variability and the lack of specific symptoms in the early stages of the disease can delay the diagnosis. In this context, several biomarkers have recently been identified as promising tools in enhancing the clinical management of PAH.

Risk assessment tools have been studied, and these greatly facilitate risk stratification and treatment decisions and prognostication in the PAH management [3]. The current therapies target three therapeutic pathways: the nitric oxide, the prostacyclin, and the endothelin pathways [3]. The paradigm for the optimal management of PAH has shifted in recent years. Upfront combination therapy with an endothelin receptor antagonist and a phosphodiesterase 5 inhibitor is now widely accepted as standard of care in PAH management [6]. In addition, there is increasing emphasis on starting prostanoids early to delay the time to clinical worsening. However, less is known regarding which prostanoid agent to employ and the optimum time to start its administration. To facilitate shared decision-making, there is an important need for decision tools based on guidelines and clinical experiences to navigate between pharmacologic and interventional treatments, as well as explore innovative, therapeutic pathways for PAH [6]. There are several promising therapies under investigation (for example, sotatercept, inhaled imatinib, ralinepag) that may further reduce morbidity and improve outcomes.

Biomarkers may serve for diagnosis, prognosis prediction, risk stratification, and dynamic monitor in patients with PAH. The aim of the present review is to summarize the role of biomarkers for the diagnosis, prognosis, and management of PAH. More specifically, the authors will try to answer the following questions: What are the properties of various biomarkers for PAH? How specific is each single biomarker? How sensitive is the detection method? How is the relationship between its content and the occurrence, development, and outcome of PAH?

## 2. Methods

To identify suitable primary research articles on this topic, an extensive online literature search on Pubmed, Embase, Clarivate Analytics/Web of Science Core Collection, and Wiley/Cochrane Library was performed up to December 2023 by using the following main keywords, alone or in logic combinations: pulmonary hypertension; pulmonary arterial hypertension, PAH; biomarkers. The date of publication was limited from 1977 to the present day. One hundred and sixteen publications were returned from this search. Only articles in English were selected; guidelines, clinical trials, reviews, and original research papers about patients affected by PAH were included. Studies were included if they reported data on biomarkers in the management of PAH patients.

Four reviewers (M.C., L.T., F. C., and E.B.) independently screened all potentially relevant titles and abstracts for eligibility. If necessary, the article full text was checked for eligibility. Differences in judgement were resolved through (1) discussions among the four reviewers; (2) arbitration by a fifth reviewer (A.P). Studies were included if they met the following criteria: (1) analyzed potential blood and urine biomarkers in any form, including growth factors, inflammatory mediators, circulating cells, proteins, (micro)RNAs, or microvesicles; (2) involved patients with group 1 PH (i.e., PAH). The following studies were excluded: (1) animal studies; (2) studies involving subjects < 18 years of age; (3) studies that did not report biomarker levels for PAH; (3) certain publication types, i.e., editorials, letters, legal cases, interviews, etc. 

## 3. Why Biomarkers in PAH?

Given the rarity of PAH (incidence and prevalence of 6 and 48–55 cases/million adults, respectively) [7], researching this patient group over a prolonged period represents a significant and continuous challenge.

Biomarkers represent ideal tools in PAH management, being capable of rapidly delivering insights into the extent of the disease or its specific traits and offering immediate information that would otherwise require extensive time to be gathered.

The search for a proper biomarker for PAH patients is still an ongoing process in the scientific community [8], with the aim of identifying novel biomarkers able to detect patients at higher risk who may require the escalation of targeted pulmonary vasodilator therapies and closer clinical surveillance. In fact, there are three main unresolved challenges in the management of patients with PAH. First, it is difficult to differentiate specific PAH etiologies. Second, invasive diagnostics are required to precisely determine the severity of PAH and, thus, to qualify patients for an appropriate treatment. Third, the results of the treatment of PAH are unpredictable and remain unsatisfactory.

Many biomarkers for PAH have been investigated, but currently we do not have a PAH-specific, easily accessible, low-priced biomarker for PAH patients. One of the reasons of that is that PAH is a disorder with a complex etiology, and the progression of this condition eventually alters multiple systems.

Moreover, given the complexity of PH, it is impossible for a single biomarker to be adequate for the broad assessment of patients with different types of PH [8]. Non-specific circulating PAH biomarkers reflect the presence of an advanced disorder with systemic hypoxemia and/or ischemia, and the levels of these biomarkers could also be elevated in many other end-stage cardiac or extracardiac conditions. Indeed, the most potentially interesting biomarkers mirror numerous pathophysiological courses [5]. Classically, biomarkers for PAH have been classified based on the pathophysiological process they reflect (i.e., markers of vascular dysfunction [asymmetric dimethylarginine (ADMA), endothelin-1, angiopoietins, etc.], markers of inflammation (C-reactive protein, interleukin 6, chemokines), markers of myocardial stress (atrial natriuretic peptide (ANP), brain natriuretic peptide (BNP)/N-terminal-proBNP (NT-proBNP), troponins), markers of low CO level and/or tissue hypoxia [pCO2, uric acid, growth differentiation factor 15 (GDF15), osteopontin], and markers of secondary-organ damage (creatinine, bilirubin) [9]. This list is constantly growing, but so far, BNP and NT-proBNP remain the only biomarkers that are widely used in routine practice in PH centers as well as in clinical trials.

However, more recent evidence suggests that a classification based on a more clinical approach represents a useful tool for disease management; as a result, the identification and the validation of reliable biomarkers for PAH are fundamental and could have a role in community screening, early diagnosis, risk stratification/prognosis, the assessment of disease severity/phenotyping, accurate management, and the monitoring of treatment response [10]. Specifically, in PAH, the use of circulating biomarkers, in addition to other non-invasive methods such as echocardiography, the assessment of NYHA/WHO functional classes, and the 6 min walking test (6MWT), could be employed to avoid useless or repeated invasive investigations such as right heart catheterization (which, however, remains the gold standard for PAH diagnosis) [11]. The identification of biomarkers that can be rapidly and cost-effectively obtained using a simple processing method still represents a challenge; indeed, despite advancements in research and the development of new drugs, mortality and the hospitalization rates of patients with PAH remain high [2], and PAH-specific markers are missing, although a wide variety of biomarkers have been explored. The lack of specific symptoms, particularly in the early stages, often leads to diagnostic delays and inevitably defers treatment initiation, leading to poorer prognoses. The combination of various biomarkers could aid in identifying, among patients with suspected PAH, those at the highest risk, warranting further investigations to ensure the best possible diagnostic and therapeutic management [12]. So, a combination of circulating, functional, and metabolic biomarkers, in addition to advanced imaging techniques and hemodynamic evaluations may provide a multidimensional approach to further enhance the clinical management of PAH.

As an additional complication, most of the circulating biomarkers described in the literature are not used in clinical practice, even though they were described years ago. For a biomarker to enter the clinical use, its utility should be consistently demonstrated in large prospective trials. However, these studies are often missing for most of the identified biomarkers [9].

In addition, several circulating biomarkers reflect the presence of an advanced disease with generalized hypoxemia and/or ischemia, and their levels correlate well with hemodynamic variables and outcomes of PAH. Nevertheless, such biomarkers could be present at elevated levels also in many other end-stage cardiac or extracardiac conditions, since they are not PAH-specific.

Therefore, future research should be focused on the identification of structural proteins specifically expressed in the pathologic tissue that act as PAH disease-specific biomarkers or on the identification of new genetic mutations. 

## 4. Role of Biomarkers in the Screening and Diagnosis of PAH

**Figure 1:**
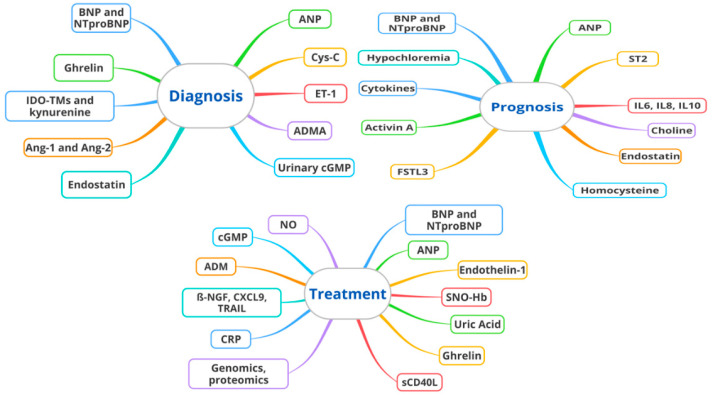
Biomarkers with a possible role in the diagnosis, prognosis, and treatment of PAH. BNP and NTproBNP (bone natriuretic factor and N-terminal pro-B-type natriuretic peptide), ANP (atrial natriuretic peptide), Cys C (cystatin-C), ET-1 (endothelin-1), ADMA (asymmetric dimethylarginine), Urinary cGMP (urinary cyclic guanosine monophosphate), Ang-1 and Ang-2 (angiopoietin-1 and angiopoietin-2), IDO-TMs (indoleamine 2,3-dioxygenase (IDO)-dependent tryptophan metabolites), ST2 (suppression of tumorigenicity 2), IL6, IL8,IL10 (interleukin-6, interleukin-8, interleukin-10), FSTL3 (follistatin-like 3), SNO-Hb (S-nitrosohemoglobin), sCD40L (soluble CD40 ligand), CRP (C-reactive protein), β-NGF, CXCL9, TRAIL (beta nerve growth factor; C-X-C motif chemokine ligand 9; TNF-related apoptosis-inducing ligand), ADM (adrenomedullin), cGMP (cyclic guanosine monophosphate), NO (nitric oxide).

Biomarkers play a crucial role in the screening and diagnosis of PAH (Table 1 and Figure 1). An early and accurate diagnosis is essential for a timely treatment. The biomarkers most commonly used for the screening and diagnosis of PAH are the natriuretic peptides (i.e., BNP, NT-proBNP, and ANP). These biomarkers are released in response to ventricular stress and/or ventricular overload and represent excellent markers of ventricular dysfunction [13]. They are the prototype of a good biomarker precisely because they satisfy all three characteristics described above (widespread and accessible; reflect disease progression and treatment effects; their measurement is reproducible).

Specifically, Nagaya et al. found that the BNP levels exhibit a positive correlation with mPAP and a negative correlation with cardiac output, highlighting a significant relationship between the BNP levels and total pulmonary resistance [14]. In scleroderma-associated PAH (SSc-PAH), the levels of NT-proBNP, an alternative biomarker to BNP, correlate with mPAP, PVR, mean right atrial pressure (mRAP), and cardiac index [15]. In addition, it was proposed as a useful tool to stratify patients with systemic sclerosis depending on the presence of PH, being included in the DETECT (DETECTion of pulmonary arterial hypertension in Systemic Sclerosis) algorithm [16].

NT-proBNP provides the same information and is still preferred to BNP in clinical practice as it has a longer half-life and greater stability and can be evaluated more accurately compared to BNP.

Similar to BNP, the atrial natriuretic peptide (ANP) levels show a positive correlation with mPAP and an inverse correlation with cardiac output. However, due to its short half-life of approximately 2 min in human subjects, BNP is considered a more suitable biomarker compared to ANP. As a result, the use of ANP is not widespread, even if the ANP levels change in response to pulmonary vasodilator therapy [17].

Even if not specific for PAH, natriuretic peptides are useful in the screening (above all, in SSc patients) and diagnosis of PAH because they are biomarkers of pressure overload, indirectly reflecting pulmonary hemodynamics and right heart impairment.

About PAH diagnosis, natriuretic peptides play a weak role; however, they have a high negative predictive value, even higher with a normal electrocardiogram; therefore, they are useful tools in ruling out PH in patients referred for suspected PH or at risk of PH.

Another useful biomarker with a possible role in PAH diagnosis is cystatin C, a renal biomarker linked to tissue hypoxia and organ damage [18]; specifically, PAH patients exhibit abnormally high CysC serum levels, which positively correlate with RV function [19]. In addition, the CysC levels are associated with elevated RV systolic pressure and show positive correlations with various RV parameters, including end-diastolic volume, end-systolic volume, mass index, strain, and strain rate [19]. Although CysC proved to be a sensitive biomarker for assessing PAH, offering advantages over standard biomarkers such as BNP and NT-proBNP by being independent of muscle mass, age, and gender [20] and predicting long-term mortality and clinical events in patients with CHD–PAH [21], its utility was demonstrated only in small studies [21]; therefore, to date, CysC has no clinical use. As a result, CysC cannot be considered a good biomarker because it is not widespread and, to the best of our knowledge, there are not data about a correlation of its expression with PAH progression.

Endothelin-1 (ET-1) promotes pulmonary artery smooth muscle cell proliferation and migration and is considered an effective vasoconstrictor. Elevated levels of ET-1 are associated with PAH [22]. ET-1 plays a key role in increased vascular tone and vascular remodeling [23]. In PAH, there is a notable upregulation of ET-1 expression in the pulmonary vasculature, particularly in plexiform lesions [22]. In addition, elevated ET-1 plasma levels are closely correlated with various PAH indicators, including RAP, pulmonary artery oxygen saturation, PVR, and 6 min walk distance [24]. Endothelial damage in PAH intensifies the constrictive effects of ET-1, leading to dysregulation in the endothelin system [25] and to a reduced capacity of the endothelium to release vasodilators [22]. This dysregulation and overexpression of ET-1 contribute to an increased pulmonary vascular resistance, partly due to a lack of vasodilators and an abnormal pulmonary vascular remodeling. High ET-1 levels are also associated with an inflammatory response and increased fibrosis [26]. The elevation in ET-1 plasma levels in PAH may result from increased ET-1 release, reduced ET-1 clearance by the pulmonary vasculature, or a combination of these mechanisms [22]. However, there are many disadvantages in using ET-1 as a biomarker in PAH patients. It can be considered a low-quality biomarker in PAH for several reasons; firstly, it is not widespread; secondly, the clinical use of ET-1 in PAH diagnosis is limited by ET-1 very short half-life (about 5 min) [27]; in addition, the ET-1 plasma levels do not accurately represent the concentration of ET-1 in tissue [22] and are affected by several confounding factors, displaying ethnic (e.g., ET-1 levels are higher in people of African ethnicity), gender (i.e., ET-1 levels are higher in males), and age (i.e., ET-1 levels are higher with ageing) differences [28].

Asymmetric dimethylarginine (ADMA), a marker of endothelial dysfunction in PAH, inhibits nitric oxide (NO) synthesis and disrupts the NO/cGMP pathway, which regulates the pulmonary vascular tone and increases vascular resistance [29]. Some studies suggest that ADMA may induce pulmonary endothelial dysfunction through changes in connexin 43 expression and activity. The ADMA levels are correlated with key hemodynamic parameters in several subgroups of PH, specifically, with mPAP, PVR, mixed venous oxygen saturation (SvO2), RAP, cardiac index, and survival in idiopathic PAH, [30], positively with RAP and negatively with SvO2, cardiac output, cardiac index, and survival rate in in congenital heart disease (CHD) PAH, [31], and with mPAP, mRAP, cardiac output, cardiac index, PVR, and SvO2 in chronic thromboembolic pulmonary hypertension (CTEPH) [31]. The ADMA levels are important for PAH early diagnosis and for monitoring disease severity, assessing therapy effectiveness, and aiding in risk stratification of patients with PAH or other types of PH [32]. In conclusion, even if its use is limited by its availability, ADMA can be theoretically considered a good biomarker of PAH.

In patients with PAH, the urinary cyclic guanosine monophosphate (cGMP) levels are elevated. Notably, these levels are particularly higher in individuals experiencing severe hemodynamic impairment, suggesting a correlation with the hemodynamic status in PAH patients [33]. Both plasma and urinary cGMP levels serve as potential indicators of hemodynamic impairment in PAH patients. However, the overall quality of the use of cGMP in PAH is poor, because cGMP is not widespread, and there are no data about its possible decrease during PAH therapy and its correlation with the hemodynamic status in patients with PAH.

Furthermore, since it reflects more of a hemodynamic condition that could also be secondary to other conditions different from PAH, we cannot consider it a specific biomarker for PAH.

The endostatin levels were found to be correlated with adverse hemodynamic changes, including higher mPAP and PVR, as well as lower cardiac index (CI) and cardiac output (CO). An elevation in Es levels was associated with poorer functional class and reduced exercise tolerance [34,35]. In the context of CHD-PAH, elevated Es levels predict worse hemodynamics and functional capacity and exhibit a direct correlation with various echocardiographic abnormalities, serving as a predictor of right ventricular dysfunction. Of note, the Es levels decrease when patients display clinical and hemodynamic improvements. Furthermore, they correlate with responses to PAH-specific treatments [36]. Es is quite far from clinical use and is not widespread.

In a recent study involving rats with monocrotaline-induced PAH, dysregulation of tryptophan metabolite (TMs) pathways was observed [37]. This dysregulation was attributed to potential metabolic reprogramming events in the pathogenesis of PAH. Specifically, the study found that in patients with PAH, the levels of IDO-TMs (indoleamine 2,3-dioxygenase metabolites), as opposed to those of tryptophan hydroxylase metabolites, were elevated and exhibited a strong correlation with right ventricular–pulmonary vasculature dysfunction [37]. This correlation encompassed resting RAP, PAP, PVR, exercise PVR, and changes in CO during exercise [38]. Furthermore, the levels of the predominant IDO-TM kynurenine not only were significantly increased in PAH patients [39,40] but also correlated with mPAP and PVR, serving as a predictor of negative patient outcomes [41]. This biomarker is very far from clinical use, being not commonly available, and it is no clear whether its levels change with treatment. We cannot consider it a specific biomarker for PAH.

Elevated total ghrelin plasma levels (the “hunger hormone”, produced in the gastrointestinal tract, which acts as an endogenous ligand, regulating energy metabolism, glucose metabolism, gastrointestinal motility, and food intake) were observed in PAH patients and associated with RV hemodynamics, specifically, with RV diameter and PAP [42]. In the context of CHD-PAH, the acyl-ghrelin levels were increased and demonstrated correlations with PAP, RV systolic pressure, mPAP, and pulmonary artery trunk diameter [43]. Additionally, the ghrelin levels were found to correlate with the NT-proBNP, ET-1, and nitric oxide (NO) levels [44,45]. In patients with atrial septal defect and PAH, the ghrelin levels were increased and exhibited a negative correlation with mPAP, suggesting that the ghrelin levels may serve as a predictor of the severity of PH in this patient population [44]. To date, ghrelin is quite far from clinical use and is not widespread. Furthermore, to our knowledge, this biomarker has not been tested in large multicenter trials. As it reflects more of one hemodynamic condition that could also be secondary to other conditions different from PAH, we cannot consider it a specific biomarker for PAH.

Most of these biomarkers have not been routinely introduced in clinical practice because several circulating biomarkers probably reflect the presence of an advanced disease with generalized hypoxemia and/or ischemia and are not PAH-specific. This makes their use for the diagnosis and screening of PAH more difficult. Therefore, it would be relevant to associate specific biomarkers with the vascular remodeling stage (early stage, with early intimal proliferation and smooth muscle hypertrophy, as opposed to late stage, with adventitial and intimal proliferation, plexiform lesion, and in situ thrombosis) of the pulmonary arterioles. The late or advanced stage of vascular remodeling may be more likely associated with generalized hypoxemia and/or ischemia and thus it with the presence of less specific biomarkers for PAH. Conversely, biomarkers associated with early vascular remodeling may be more specific for PAH.

Future research on the diagnosis of PAH should be focused on the identification of structural proteins specifically expressed in the pathologic tissue that act as PAH disease-specific biomarkers. Genetic screening of patients at high risk of developing PAH (SSc, CHD, HIV, advanced liver disease patients) should be considerably increased and enriched by the discovery of new genes or new mutations associated with this syndrome.

## 5. Biomarker-Guided Therapies for PAH

Predicting the clinical response to a given treatment is one of the most difficult challenges in PAH pharmacotherapy. The identification of a treatment-relevant biomarker could enhance decision-making and potentially improve outcomes in patients with PAH. To date, the only predictive biomarker employed for selecting PAH therapy is vasoreactivity; specifically, patients who exhibit positive vasoreactivity (i.e., acute vasodilation in the presence of inhaled nitric oxide during cardiac catheterization) are candidates for calcium channel blocker therapy [46].

However, several drugs are now available, and several circulating biomarkers related to the pathways targeted by these drugs could have a potential role in guide PAH treatment.

### 5.1. Endothelin Pathway

ET-1 is a potent vasoconstrictor involved in the pathogenesis of PAH; as a result, the endothelin pathway represents a therapeutic target that can be impacted by specific medications. Endothelin receptor antagonists (ERAs) have proven effective in treating PAH by inhibiting endothelin receptors, reducing PAP, and counteracting vascular remodeling. Macitentan, the newest drug in this class, improved mPAP, RAP, PVR, CI, and the levels of NT-proBNP [47]. ET-1 could be considered an ideal prognostic marker of disease progression, its levels correlating with responses to PAH-specific treatments.

### 5.2. Nitric Oxide and Cyclic Guanosine Pathway

Nitric oxide synthase produces nitric oxide (NO) from L-arginine, initiating cGMP production through soluble guanylate cyclase (sGC), leading to vascular smooth muscle relaxation and vasodilation [48,49]. Additionally, more stable products, S-nitrosothiols (SNOs), regulate cardiovascular and pulmonary functions by influencing protein activity [50,51]. In advanced PAH patients, a study showed reduced vasodilation capacity in red blood cells (RBCs) under low-oxygen conditions, accompanied by lower levels of RBC S-nitrosohemoglobin (SNO-Hb) [52]. The inhalation of ethyl nitrite, releasing SNOs, significantly improved pulmonary arterial pressure and vascular resistance, enhancing RBC-related vasodilation and increasing the RBC SNO levels. This suggests that SNO-Hb may serve as a valuable biomarker in clinical settings for assessing and managing PAH [53]. However, to our knowledge, this biomarker has not been tested in large multicenter trials and is far from clinical use.

It was hypothesized that exhaled nitric oxide (FeNO) and exhaled breath temperature (EBT) may have a potential role as biomarkers in patients with PH (EBT reduction was correlated with PAPm increase, whereas the levels of FeNO were higher in PH-COPD patients) [54].

Specifically, several studies indicated increased levels of exhaled NO in PAH patients following treatment with Bosentan [55] and Epoprostenol (a prostacyclin analog) [56,57]. Moreover, the use of phosphodiesterase (PDE) inhibitors like Zaprinast demonstrated an increase in vasodilator responsiveness to inhaled NO in the lungs of rats challenged with endotoxin (lipopolysaccharide) [58]. While NO alone does not seem to be useful as a biomarker for PAH (also because it does not seem specific for PAH), when combined with additional data, it may be valuable in understanding patients’ responses to therapy with prostacyclin and Bosentan [55]. The use of FeNO is not widespread in clinical practice. To our knowledge, this biomarker has not been tested in large multicenter trials.

Studies also emphasize the significance of adrenomedullin (ADM) as a therapeutic target for PAH. The administration of exogenous ADM led to substantial hemodynamic improvements, including an increase in CI and a decrease in PVR in PAH patients [59]. Beyond its therapeutic role, ADM acts as a disease-regulating hormone in PAH and serves as an alternative marker for prognosis and disease severity [60]. However, ADM is absolutely not widespread in clinical practice. Reflecting more of one hemodynamic condition that could also be secondary to other conditions different from PAH, we cannot consider it a specific biomarker for PAH.

### 5.3. Inflammation

Inflammation is a characteristic of PAH, with high levels of circulating cytokines (IL-1β, IL-6, and TNF-α) involved in the initiation and progression of PAH. Some studies suggested that biomarkers tied to inflammatory and immune processes may play a role in PAH, providing insights into the complex interaction between immune response and pulmonary vascular remodeling [61]. Increased levels of pro-inflammatory serum cytokines (e.g., interferon-gamma; interleukin (IL)-1beta, -2, -4, -5, -6, -8, -10, -12p70, and -13; tumor necrosis factor-alpha (TNF–α)) were demonstrated in patients with IPAH and HPAH compared to the control group. The CXCL9 (chemokine), nerve growth factor β (β-NGF), and TNF-related apoptosis-inducing ligand (TRAIL) levels were independently associated with prognosis both at the time of PAH diagnosis and at the first follow-up after initiation of PAH therapy. It was demonstrated that the levels of these biomarkers change with disease progression; therefore, the monitoring of the β-NGF, CXCL9, and TRAIL levels in the serum should be considered to improve the management and treatment of these patients [61]. The serum-soluble TRAIL levels decreased in patients who responded to treatment [62] and could be a biomarker for diagnosis and effective therapy for PH patients. The main limitation of using the β-NGF, CXCL9, and TRAIL levels is that their use is not widespread in clinical practice.

C-reactive protein (CRP), a member of the pentraxin family of proteins, is predominantly synthesized in hepatocytes in response to cytokines such as interleukin (IL)-6 and IL-1 and is a sensitive marker of underlying systemic inflammation. In addition to being prevalent in inflammation, CRP also plays a key role in endothelial dysfunction, atherosclerosis, and cardiovascular diseases, having been recognized as a risk predictor of cardiovascular events [63]. Quarck et al. revealed that elevated CRP levels in PAH patients not only correlate with RAP, NYHA functional class, 6MWT, and survival but also serve as a predictor of outcomes and therapeutic response. Additionally, their findings showed that effective treatments that stabilize the CRP plasma levels in PAH patients are associated with a significantly higher survival rate, accompanied by classification in a less severe NYHA functional class and an increase in CI [64]. The CRP levels could be used for establishing patient prognosis, as well as for evaluating the therapeutic response in PAH patients. CRP is quite widespread and very accessible. Its measurement is reproducible. About its quality as a biomarker, CRP may be a good biomarker. However, it cannot be considered a specific biomarker for PAH and is not useful in the diagnosis of PAH.

The main limitation of using the CRP levels and the presence of D-dimers as biomarkers is that they can be elevated due to inflammatory (CRP levels and D-dimers) or procoagulatory states (D-dimers) of various etiologies; therefore, they have very low specificity for diagnostic purposes [65]. However, they may be useful for evaluating disease severity.

CD40 is a type I transmembrane receptor belonging to the TNF superfamily of receptors [66]. Its ligand (CD40L) and its soluble form (sCD40L) have immunomodulating activity and belong to the TNF superfamily. The interaction of CD40 with CD40L, in its soluble and/or transmembrane form, induces the activation of inflammatory and coagulation pathways in the vascular endothelium and promotes the activation of immune and non-immune cells [67]. Damås et al. [68] found elevated sCD40L levels in PAH patients, proposing that the CD40-CD40L interaction contributes to increased chemokine expression. Recombinant sCD40L induced IL-8 and MCP-1 production in endothelial cells, correlating with the sCD40L levels and hemodynamic parameters in PAH patients. Platelets from PAH patients exhibited increased sCD40L release [68]. Another study suggested that turning off the CD40 pathway in endothelial progenitor cells, a potential therapy for PAH, may enhance the efficacy of transplantation [69]. In the literature, there are few studies where the sCD40L levels were tested in PAH patients. sCD40L is not specific for PAH.

### 5.4. Hypoxia/Organ and Tissue Damage

Although uric acid (UA), the end-product of adenine oxidation and guanine purine metabolism, is not specific for PAH (in situations of hypoxia, ischemia, chronic HF, cyanotic congenital heart disease, and COPD, the UA levels are increased as a reflection of compromised oxidative metabolism), some association with PAH parameters were demonstrated. Specifically, Nagaya et al. [66] found that patients with PAH exhibit elevated levels of UA compared to controls. Additionally, the serum UA levels positively correlate with a more severe NYHA functional class, total PVR, and mortality, while negatively correlate with CO. The UA levels are elevated and correlated with disease severity in PAH patients. Furthermore, vasodilator therapy is associated with a decrease in the UA levels, in parallel with a reduction in total PVR [70]. Therefore, it was suggested that UA can be used as a biomarker for monitoring treatment during vasodilator therapy. UA is very widespread and accessible. Its measurement is reproducible. However, increased UA levels are dependent on age and sex, and the UA values are affected by renal activity, since approximately two-thirds of UA is excreted through the kidneys and one-third of it through the gastrointestinal tract. In addition, the main limitation of UA as a marker is the lack of specificity for PAH.

Many circulating biomarkers probably reflect the presence of an advanced disease with generalized hypoxemia and/or ischemia and are not PAH-specific. These biomarkers are consequently associated with more advanced disease and correlate well with hemodynamic variables and with outcomes in PH patients. In this context, activin A appeared to be a more specific biomarkers for pulmonary vascular disease, able to identify a worse prognosis specifically in the presence of PAH, serving as a guidance for treatment or even as a therapeutic target. This is due to its correlation with the TGFB pathway and, subsequently, with sotatercept treatment. Sotatercept is a soluble fusion protein comprising the extracellular domain of activin receptor type IIA linked to the Fc portion of human IgG1 and is a first-in-class activin signaling inhibitor under development for the treatment of PAH [71]. In our opinion, in the therapeutic choice, it would be safer and at the same time more precise to adopt an integrated approach involving biomarkers and imaging.

## 6. Role of Biomarkers in the Prognosis of PAH

Several biomarkers have been studied as prognostic tools for patients with PAH (Table 1 and Figure 1). In this regard, natriuretic peptides (BNP and NT-proBNP) and serum cardiac troponins have been well established as indicators of right ventricular dysfunction and disease severity. Both BNP and NT-proBNP are included in the REVEAL Registry risk score and the REVEAL 2.0 [72,73]. Their prognostic value has also been pointed out by Hendriks et al. in a systematic review and meta-analysis, showing a strong correlation between elevated NT-proBNP levels at the time of diagnosis and mortality or lung transplant [74]. Soluble suppressor of tumorigenesis 2 (ST2) protein is another promising biomarker for PH patients. ST2 belongs to the toll–interleukin 1 receptor superfamily and exists in two isoforms, i.e., as a transmembrane ST2 ligand and as a soluble form that circulates in the blood. The transmembrane form is expressed mainly on inflammatory cells and takes part in the strengthening of the immune response of Th2 lymphocytes. However, it is also exposed in cardiomyocytes and the endothelium. Together with BNP, soluble ST2, a regulator of inflammation, proved to be significantly associated with PAH hemodynamic parameters (i.e., RAP, mPAP, PVR, and RV stroke work) [45], 6MWT, NT-proBNP, and mortality [75]. ST2 could reflect PAH severity, with a sensitivity and specificity of 83.3% and 78.6%, respectively. Increased levels of ST2 may be also showed in other disease such as heart failure, myocardial infarction, or acute aorta dissection [76] (therefore, ST2 cannot be considered specific for PAH). ST2 constitutes a general biomarker of PH regardless of the subtype (in different types of PH, higher sST2 levels are linked to the remodeling of the RV); instead, growth differentiation factor-15 (GDF-15 or macrophage-inhibiting cytokine), a stress-responsive member of the TGFβ cytokine superfamily, is a specific biomarker of postcapillary PH. It is secreted in response to oxidative stress, inflammation, hypoxia, telomere erosion, and oncogene activation. In PH, GDF-15 is found in plexiform lesions in the pulmonary vascular bed, affecting both apoptosis and proliferation of endothelial cells. Moreover, it was shown to be elevated in patients with IPAH. Increased levels were also demonstrated in patients with SSC–PAH and positively correlated with PVR and the plasma NT-proBNP value. In patients with SSc-PAH, GDF-15 was a marker of reduced survival and correlated with right ventricular systolic pressure assessed by transthoracic echocardiography. There were no significant changes in the GDF-15 values after specific therapy at a three- or six-month follow-up. However, both GDF-15 and ST-2 are quite far from a widespread use in the PAH management.

In addition, reflecting the importance of inflammation in this pathology, also circulating cytokines such as IL-6, IL-8, and IL-10 were associated with low survival rates in IPAH and HPAH patients, suggesting their possible role in risk-stratifying patients, as for other CV diseases [77]. However, because of their low sensitivity and specificity, they are far from being routinely used in clinical practice.

The sensitivity of the current ESC/ERS risk assessment strategy may be improved by the addition of biomarkers related to PAH pathophysiology. However, most of these biomarkers have not been validated in large trials and are therefore not adopted in clinical practice.

Serum cardiac troponins (cTnI and cTnT) can be detected with high sensitivity. Troponins, widespread and accessible biomarkers, are markers of advanced disease, and the interpretation of the troponin levels in PAH patients is strongly influenced by their association with renal failure or left-sided heart failure. In patients undergoing treatment for PH, it was found that the cTnT levels decreased; in contrast, the levels increased with the progression of the disease [78]. Clinical studies highlighted a direct correlation between plasmatic troponin levels and increased PVR, lower RV ejection fraction, lower mixed venous oxygen saturation (mvSatO2), and shorter 6MWT. Even though they are not specific for PAH, troponins can be considered a good biomarker for PAH.

A growing body of evidence is showing the association of metabolites deriving from several metabolic pathways with cardiovascular diseases [79]. For instance, choline, a precursor of microbiota-derived metabolites that promote vascular ageing, whose role was already demonstrated in hypertension, atherosclerosis, myocardial infarction [80], and heart failure [81,82], was investigated as a biomarker of PH of various etiologies. Specifically, high baseline choline levels (representative of a western diet [83]) were significantly associated with adverse outcomes in PAH patients, as well as with elevated NT-proBNP levels, higher WHO-FC, and a low cardiac output index [84]. However, they are far from being used in clinical practice, and no data are available with regard to the correlation between the levels of these markers and disease progression during specific PAH therapy.

Homocysteine, a sulfur-containing intermediate product of the normal metabolism of methionine, an essential amino acid, is obtained essentially from animal protein, and its synthesis requires choline; it is a well-known cardiovascular risk factor. The increase in homocysteine levels, known as hyperhomocysteinemia, results in a decrease in NO bioavailability. This decrease in NO is linked to the inhibition of the activity of dimethylarginine dimethylaminohydrolase by homocysteine. This inhibition promotes the accumulation of ADMA and, consequently, a decrease in the production of NO by endothelial cells.

It could be used as a non-invasive prognostic parameter in PAH. Indeed, Wang M. et al., after grouping patients into three classes of risk according to the ESC/ERS guidelines, pointed out that the homocysteine levels had a linear correlation with the risk class and the NT-proBNP levels and an inverse correlation with DLCO (an independent risk factor for survival) [85]. However, no data are available about the pharmacological response to specific pulmonary vasodilators.

Since changes in microvascular vessels are linked to right ventricle perfusion and remodeling in PAH, some researchers studied the role of serum endostatin, an important angiostatic peptide, in disease prognosis. They found a positive correlation between the Es levels and mPAP, RAP, PVR, NT-proBNP, and the NYHA class and a negative correlation with cardiac output and cardiovascular performances in the 6MWT. Moreover, high Es levels were predictive of adverse outcomes with an unadjusted hazard ratio of 4.3 [35] However, because of a low specificity, Es is not currently used in clinical practice.

Serum chloride represents a simple and available biomarker able to identify patients at high risk of PAH. Indeed, hypochloremia is involved in the mechanism of diuretic resistance that worsen volume overload, leading to right heart dysfunction [86]. Specifically, low serum chloride levels were associated with higher BNP levels, RV dysfunction on echocardiogram, and higher right atrial pressures. Lastly, this dyselectrolytemia is associated with reduced 6MWD and increased mortality [87]. However, such biomarkers (e.g., uric acid, chloride) could also be elevated in many other end-stage cardiac or extracardiac conditions and, therefore, they are absolutely nonspecific for PAH.

Bone morphogenetic protein (BMP) receptor type II (BMPRII) is a receptor involved in transforming growth factor beta (TGF-β) signaling and is expressed in the pulmonary vascular endothelium and in smooth muscle cells [88]. An imbalance between the BMPRII and TGF-β pathways is implicated in PAH predisposition and pathogenesis. In fact, disturbed BMP/TGF-β signaling impedes blood vessel formation and impairs vascular integrity, resulting in the development of vascular damage, including PH [89].

Mutations or maladaptation of regulators and component proteins in the BMP/TGF-β axis is implicated in the development of PAH [90]. Animal studies implicate loss of function in BMP signaling and maladaptive TGF-β signaling as drivers of PAH. Activins represent a family of TGFβ-like proteins that were shown to play a role in a variety of developmental pathways. Activins are dimeric proteins composed of β_A_- and β_A_-subunit (activin A), β_B_- and β_B_-subunits (activin B), or β_A_- and β_B_-subunits (activin AB). Activin effects are modified (in part) by activin-binding protein (follistatin) and inhibin [91].

Although sharing common receptors and effectors with BMP/TGFβ, the role of activin is not completely understood. Some studies reported of elevated circulating levels of activin A and follistatin in PAH patients [92]. Recently, activin A and its antagonist follistatin-like 3 (FSTL3) were found to be prognostic biomarkers of PAH [93]

Kumpers et al. found that in patients with IPAH, the venous plasma levels of angiopoietin -1 (Ang-1) and angiopoietin-2 (Ang-2) were elevated. Notably, only Ang-2 demonstrated significant correlations with multiple indicators of poor prognosis, including mRAP, PVR, NYHA functional class, cardiac index, and SvO2. Furthermore, Ang-2 emerged as a significant predictor of mortality, and its levels exhibited an inverse relationship with changes in mRAP, PVR, and SvO2 following the initiation of treatment [94]. Another study reinforced these findings, indicating that elevated Ang-2 levels in IPAH patients decreased in tandem with improvements in the 6 min walk distance (6MWD) during Treprostinil monotherapy [95]. As a result, Ang-2 could be used to monitor the pharmacological response; however, it is not widespread and is not used in clinical practice.

The plasma levels of proteins related to tumor necrosis factor (TNF), inflammation, and immunomodulation were analyzed in patients with PAH, chronic thromboembolic pulmonary hypertension, PH due to left heart failure (HF) with preserved (HFpEF-PH) or reduced (HFrEF-PH) ejection fraction, HF without PH, and healthy controls. Specifically, the TRAIL levels were lower in PAH patients versus patients with other diseases and controls [96]. In receiver operating characteristics analysis, the TRAIL levels identified PAH patients from those in other disease groups. TRAIL identified PAH patients among patients with dyspnea and differentiated them from those with CTEPH, HF with and without PH, and healthy controls. [96] TRAIL could be useful for the diagnosis of PAH, but it is not widespread and is usually not used in clinical practice. Also, in the prognostic stratification of PAH patients, we support the idea that an integrated approach of imaging and biomarkers would be the best and safest approach.

## 7. Future Developments

In PAH patients, future biomarker research should be primarily directed to finding biomarkers linked to novel pharmacological targets for new drugs. A growing body of studies using a proteomic [97] or metabolomic [98] approach is ongoing. Specifically, several studies explored the potential of omics techniques (genomics, proteomics, etc.) in predicting how patients with PAH may respond to specific therapies.

For instance, the use of functional and metabolic biomarkers was recently explored [99], including biomarkers of mitochondrial dysfunction and oxidative uncoupling.

A lack of biomarkers to differentiate specific PAH etiologies and evaluate the severity of PAH, as well as the small number of pharmacologic targets in PAH offer the possibility to use miRNAs in PAH management. MicroRNAs (miRs) are small non-coding RNAs that regulate post-transcriptional gene expression. Their role as prognostic and diagnostic biomarkers in many different diseases has been studied in recent years [100,101,102]. Exosomes containing miRs have emerged as novel biomarkers. Transpulmonary exosomal miRs offer valuable insights into pulmonary circulation microenvironments [103]. A relevant expression of transpulmonary exosomal miR-21 may be demonstrated in patients with CHD-related PAH [103]. Further investigation is warranted to elucidate the regulatory mechanisms involving miR-21 in the pathophysiology of PAH.

miR-8078 is upregulated in CHD-PAH patients, and bioinformatic analysis indicated its involvement in the pathogenesis of CHD-PAH, suggesting its role as a potential therapeutic target or biomarker [104].

Hemnes and colleagues conducted a genetic analysis on 36 PAH patients and identified around 1500 new genetic variants [105]. They observed more rare gene variants in PAH patients who responded to vasodilators compared to non-responders, suggesting the presence of distinct genetic factors between these groups. Notably, the responders exhibited an overrepresentation of genes linked to vascular smooth muscle contraction. In a broader analysis involving 715 PAH patients treated with endothelin receptor antagonists, specific genetic variants, particularly associated with the G protein complex genes GNG2 (G protein subunit gamma 2) and GNAS (guanine nucleotide-binding protein, alpha stimulating), showed strong associations with clinical outcomes [106].

Future research can be focused on the hypothesis that biomarkers can help clinicians to phenotype patients suffering from different forms of PH (e.g., interstitial lung disease, chronic obstructive pulmonary disease with PH [107,108], and advance HF with PH) and possibly direct them to specific drugs other than pulmonary vasodilators [109].

Recently, some authors investigated the role of several cytokines for PAH prognosis [62]. These authors found that some biomarkers of inflammation and/or fibrosis (B-NGF, CXCL9, and TRAIL) could be more important than many other clinical parameters. These authors evaluated the biomarkers’ levels at baseline and during follow-up and validated their results externally [62]. As these biomarkers are emerging as the most relevant cytokines among many others investigated and could be even more informative than other extended clinical parameters (functional class, 6MWT, RAP, and CI) we can consider them as one of the main breakthroughs in this field. Further research in this field should be developed in the near future. A possible mean to overcome the lack of specific PAH biomarkers could be that of following a multimarker strategy. However, there are still insufficient data on the application of these strategies for the monitoring and risk stratification of PH patients.

Finally, a few studies examined different biomarkers for RV dysfunction detection in the perioperative setting, and their validation is warranted before clinical application [110]. We hope for further research in this direction.

## 8. Limitations of the Current Evidence

Several are the limitations of the currently available literature on the topic.

Firstly, a limitation of biomarker research arises from the rarity of PAH (PAH incidence and prevalence are of 6 and 48–55 cases/million adults, respectively) and the heterogeneity of PAH patients (HIV, congenital heart disease (CHD), systemic sclerosis patients). Secondly, the mechanisms underlying the development of different PH subgroups are not the same (for instance, CHD patients widely differ from patients with IPAH or connective tissue diseases). In addition, the progression of this condition eventually alters multiple systems. Moreover, given the complexity of PH, it is impossible for a single biomarker to be adequate for the broad assessment of patients with different types of PH [8]. Non-specific circulating PAH biomarkers reflect the presence of an advanced disorder with systemic hypoxemia and/or ischemia, and the levels of these biomarkers could also be elevated in many other end-stage cardiac or extracardiac conditions. Another issue derives from the low availability of most biomarkers described in the present review. Furthermore, there are no data with regard to the association between biomarker changes and the pharmacological response to specific pulmonary vasodilators. So, a combination of circulating, functional, and metabolic biomarkers, in addition to advanced imaging techniques and hemodynamic evaluations, may provide a multidimensional approach to further enhance the clinical management of PAH. However, there are still insufficient data on the application of these strategies for the monitoring and risk stratification of PH patients. Future research should be focused on the identification of structural proteins specifically expressed in the pathologic tissue that act as PAH disease-specific biomarkers. Finally, there is a good chance that exosomes containing miRs will become specific biomarkers.

## 9. Conclusions

Biomarkers of PAH play an important role in patient screening, disease diagnosis, and treatment monitoring. They are even more relevant for risk stratification/prognosis. Furthermore, the search of new biomarkers of PAH could lead to the discovery of new pharmacological targets for new drugs. In addition, biomarkers can help clinicians to phenotype patients suffering from different forms of PH and possibly direct them to drugs other than specific pulmonary vasodilators. However, to date, research is still far from being able to phenotype the various forms of PH, and numerous studies are still needed to better understand the pathophysiology of PH.

Actually, it is hard to recognize a PAH-specific and easily accessible biomarker for PAH patients. The search of a proper biomarker for PAH patients is an ongoing process.

In our opinion, future research should be focused on the identification of structural proteins specifically expressed in the pathologic tissue that act as PAH disease-specific biomarkers. 

## Figures and Tables

**Table 1:** Biomarkers with a possible role in the PAH continuum.

Diagnosis	Prognosis	Treatment
BNP and NTproBNP	BNP and NTproBNP	BNP and NTproBNP
ANP	ANP	ANP
Cystatin C	ST2	Endothelin-1
Endothelin-1	IL6, IL8, IL10	SNO-Hb
ADMA	Choline	NO
Urinary cGMP	Homocysteine	cGMP
Ang-1 and Ang-2	Endostatin	ADM
Endostatin	Hypochloremia	ß-NGF, CXCL9, TRAIL
IDO-TMs, kynurenine	Cytokines	CRP
Ghrelin	Activin A	Uric Acid
	FSTL3	Ghrelin
		sCD40L
		Genomics, proteomics
